# IL-13 but not IL-4 signaling via IL-4Rα protects mice from papilloma formation during DMBA/TPA two-step skin carcinogenesis

**DOI:** 10.1002/cam4.145

**Published:** 2013-10-22

**Authors:** Michael Rothe, David Quarcoo, Anna A Chashchina, Svetlana V Bozrova, Zhihai Qin, Sergei A Nedospasov, Thomas Blankenstein, Thomas Kammertoens, Marina S Drutskaya

**Affiliations:** 1 Institute of Immunology, Charité Campus Buch13125, Berlin, Germany; 2 Institute of Occupational Medicine, Charité Campus Benjamin FranklinThielallee 69-73, 14195, Berlin, Germany; 3 Engelhardt Institute of Molecular Biology, Russian Academy of Sciences32 Vavilov Str., 119991, Moscow, Russia; 4 Biological Faculty, Lomonosov Moscow State University119991, Moscow, Russia; 5 Institute of Biophysics, Chinese Academy of Sciences15 Datun Road, Beijing, 100101, China; 6 Max-Delbrück-Center for Molecular Medicine13125, Berlin, Germany

**Keywords:** Carcinogenesis, DMBA TPA, IL-13, IL-4 receptor alpha, papilloma, TH2

## Abstract

Interleukin 4 (IL-4) was shown to be tumor-promoting in full carcinogenesis studies using 3-methylcholanthrene (MCA). Because heretofore the role of IL-4 in DMBA/TPA (9,10-dimethyl-1,2-benz-anthracene/12-*O*-tetradecanoylphorbol-13-acetate) two-stage carcinogenesis was not studied, we performed such experiments using either IL-4^−/−^ or IL-4Rα^−/−^ mice. We found that IL-4Rα^−/−^ but not IL-4^−/−^ mice have enhanced papilloma formation, suggesting that IL-13 may be involved. Indeed, IL-13^−/−^ mice developed more papillomas after exposure to DMBA/TPA than their heterozygous IL-13-competent littermate controls. However, when tested in a full carcinogenesis experiment, exposure of mice to 25 μg of MCA, both IL-13^−/−^ and IL-13^+/−^ mice led to the same incidence of tumors. While IL-4 enhances MCA carcinogenesis, it does not play a measurable role in our DMBA/TPA carcinogenesis experiments. Conversely, IL-13 does not affect MCA carcinogenesis but protects mice from DMBA/TPA carcinogenesis. One possible explanation is that IL-4 and IL-13, although they share a common IL-4Rα chain, regulate signaling in target cells differently by employing distinct JAK/STAT-mediated signaling pathways downstream of IL-13 or IL-4 receptor complexes, resulting in different inflammatory transcriptional programs. Taken together, our results indicate that the course of DMBA/TPA- and MCA-induced carcinogenesis is affected differently by IL-4 versus IL-13-mediated inflammatory cascades.

## Introduction

The first experimental carcinogenesis protocols were established a century ago by Yamagiva and Ichikawa [1], who painted coal tar on rabbit ears. Subsequent skin carcinogenesis studies showed that tumor development after a single sub-tumorigenic dose of a mutagen can be induced and enhanced by a tumor promoter, such as croton oil, that is not tumorigenic by itself [2]. While the mutagen/carcinogen interacts with the DNA to cause mutations, the tumor promoter is thought to cause epigenetic changes in the mutated cells either directly or indirectly via the microenvironment. Various polycyclic aromatic hydrocarbon compounds can be used to initiate mutations for the induction of skin cancer (e.g., 9,10-dimethyl-1,2-benz-anthracene/DMBA, benzopyrene/BaP, methylcholantrene/MCA). Other compounds can be used to enhance/promote tumor formation (e.g., croton oil, 12-*O*-tetradecanoylphorbol-13-acetate/TPA, or okadaic acid). To date, one of the most frequently used two-step skin carcinogenesis models involves application of the carcinogen DMBA followed by repeated applications of the tumor promoter TPA [3, 4]. Skin carcinogenesis studies helped to establish paradigms of initiation, promotion, and progression of cancer. Various factors affecting chemical skin carcinogenesis have been described, such as wounding [5], diet [6, 7], composition of skin surface lipids [8], glucocorticoid levels [9], and genetic background [4, 10]. Because tumor promoters, such as TPA, cause a local inflammatory response, it is not surprising that immune cells and inflammatory processes can influence skin carcinogenesis. For example, experiments in mice lacking αβ-T cells or γδ-T cells suggested that αβ-T cells are tumor-promoting [11–14], while γδ-T cells appeared to be protective [11]. The tumor-promoting subset of αβ-T cells seems to vary depending on the genetic background. While in the FVB (an inbred strain of mice) genetic background CD8^+^ T cells are thought to be more important in tumor promotion [13], in the C3H background, CD4^+^ T cells are thought to be tumor-promoting while CD8^+^ T cells are considered to be protective [14]. Furthermore, cytokines such as interferon γ (IFN-γ) and interleukin 12 (IL-12) have both been shown to promote tumor development [15, 16], and systemic administration of recombinant mouse IFN-γ resulted in increased numbers of papillomas [17]. In terms of cytokine signaling, it has been shown that STAT3, a signal transducer involved in regulation of various cytokines (e.g., IL-5, IL-6, interferons, and leukemia inhibitory factor), contributes to carcinogenesis [18]. In addition, the transcription factor NF-κB, a downstream effector of tumor necrosis factor α (TNF-α), has been suggested to affect skin carcinogenesis [19]. While it is known that IL-4 also involves NF-κB (nuclear factor kappa light-chain enhancer active in B cells) signaling, the effect of this type 2 helper T cells (TH2)-cytokine as well as the role of (TH2)-dependent inflammatory responses, in general, in skin carcinogenesis is incompletely understood. As TH2 cytokines have been associated with tissue responses like wound healing and fibrosis, which are both implicated in tumorigenesis, it is reasonable to assume that TH2 cytokines may have an impact on skin tumor development. Hence, we addressed the role of IL-4 and IL-13 in two-step skin carcinogenesis in this study.

## Material and Methods

### Mice

IFN-γ-deficient (IFN-γ^−/−^) mice on the BALB/c background (F6) and wild-type BALB/c mice were purchased from the Jackson Laboratories (Bar Harbor, ME). IL-4-deficient (IL-4^−/−^), IL-4 receptor α chain-deficient (IL-4Rα^−/−^) mice were generated with BALB/c embryonic stem cells, recombination activating gene 2-deficient (RAG2^−/−^) and RAG2^−/−^/IL-4Rα^−/−^ double-deficient mice were kindly provided by N. Noben-Trauth. IL-13-deficient (IL-13^−/−^) mice were obtained from the MRC Laboratory of Molecular Biology, Cambridge, and were backcrossed to the BALB/c genetic background for six generations. Heterozygous and homozygous gene-deficient mice were generated by intercrossing gene-deficient mice first with the respective control strain (e.g., BALB/c) and afterward crossing the heterozygous F1 mice back to the respective knockout mice. The offspring were genotyped using the primers suggested by the Jackson Laboratories. All mice were bred at the animal facility of the Max Delbrück Center, Berlin.

### Tumor induction

For induction of papillomas with DMBA/TPA a dorsal area of skin of ∼4 cm^2^ was shaved and 20 μg of DMBA dissolved in 100 μL acetone was administered by a micropipette. Starting 1 week later, 4 μg TPA dissolved in 100 μL acetone was applied on the same area of the skin three times weekly for 20 weeks. The area was regularly depilated and tumors were assessed two times weekly and were defined as raised lesions of a minimum diameter of 1 mm that had been present for at least 1 week. Mice were sacrificed if the tumor size reached ≥10 mm in diameter or if tumors became invasive or ulcerated.

For induction of tumors with MCA, mice were injected s.c. in the left abdominal region with 25 μg MCA in 0.1 mL of sesame oil. Tumor development was monitored two to three times weekly. Mice with a tumor size of ≥10 mm in three perpendicular diameters were counted as tumor positive.

### Quantitative reverse transcriptase polymerase chain reaction

Total RNA was isolated from 10 mm by 10 mm piece of shaved back skin using TRIzol reagent (Ambion, Life Technologies, Carlsbad, CA). Prior to reverse transcription 1 μg of RNA was treated with RNase-free DNase I (Thermo Scientific, Waltham, MA). Reverse transcription was performed on 330 ng total RNA, random hexamers primed with SuperScript® III First-Strand Synthesis System for reverse transcriptase polymerase chain reaction (RT-PCR; Invitrogen [Carlsbad, CA], Life Technologies). Quantitative PCR amplifications were performed from 50 ng of cDNA using Power SYBR Green Master Mix (Applied Biosystems, Life Technologies) and Qiagen (Hilden, Germany) primer sets to murine GAPDH (Cat.# PPM02946E), IL-4Ra (Cat.# PPM03025F), IL-4 (Cat.# PPM03013F), IL-13 (Cat.# PPM03021B), IL-13Ra1 (Cat.# PPM03133F), IL-13Ra2 (Cat.# PPM03556C), STAT6 (Cat.# PPM04646F), IFN-γ (Cat.# PPM03121A), IFN-γR1 (Cat.# PPM03134F). Amplifications were performed on StepOne Real-Time PCR Instrument (Applied Biosystems, Foster City, CA) as follows: one cycle for 10 min at 95°C, 40 cycles at 95°C for 15 sec, annealing 60°C for 1 min, and an extension of 90 sec at 72°C.

### Cytometric bead array for serum cytokine analysis

Blood samples were allowed to agglutinate followed by two consecutive centrifugations (20,000*g*, 10 min, 4°C). Supernatant was transferred into a fresh tube after each centrifugation. Serum samples were aliquoted and stored at −20°C. Aliquots were used only once to determine serum levels of cytokines. Thirty microliters of serum was analyzed using the cytometric bead array from Bender Medsystems (eBioscience, San Diego, CA) according to the manufacturer's instructions. From this sample, the levels of the following 10 cytokines were determined using the TH1/TH2 10-plex kit (respective detection limits): IL-1α (15.7 pg/mL), IL-2 (8.8 pg/mL), IL-4 (0.7 pg/mL), IL-5 (4.0 pg/mL), IL-6 (2.2 pg/mL), IL-10 (5.4 pg/mL), IL-17 (2.4 pg/mL), IFN-γ (6.5 pg/mL), TNF-α (2.1 pg/mL), and granulocyte macrophage colony stimulating factor (GM-CSF) (10.9 pg/mL). Flow cytometric analysis of samples was performed on a BD FACS Calibur and data were analyzed using the Bender Medsystems software.

### Ethics statement

Animal experiments were performed with approval of the Landesamt für Gesundheit und Soziales, Berlin, Germany.

## Results

### TPA treatment enhances local IL-4Rα, IL-13, IL-4, and IL-13Rα1 mRNA expression in the skin and enhances systemic levels of IL-5 and IL-6

**Figure 1:**
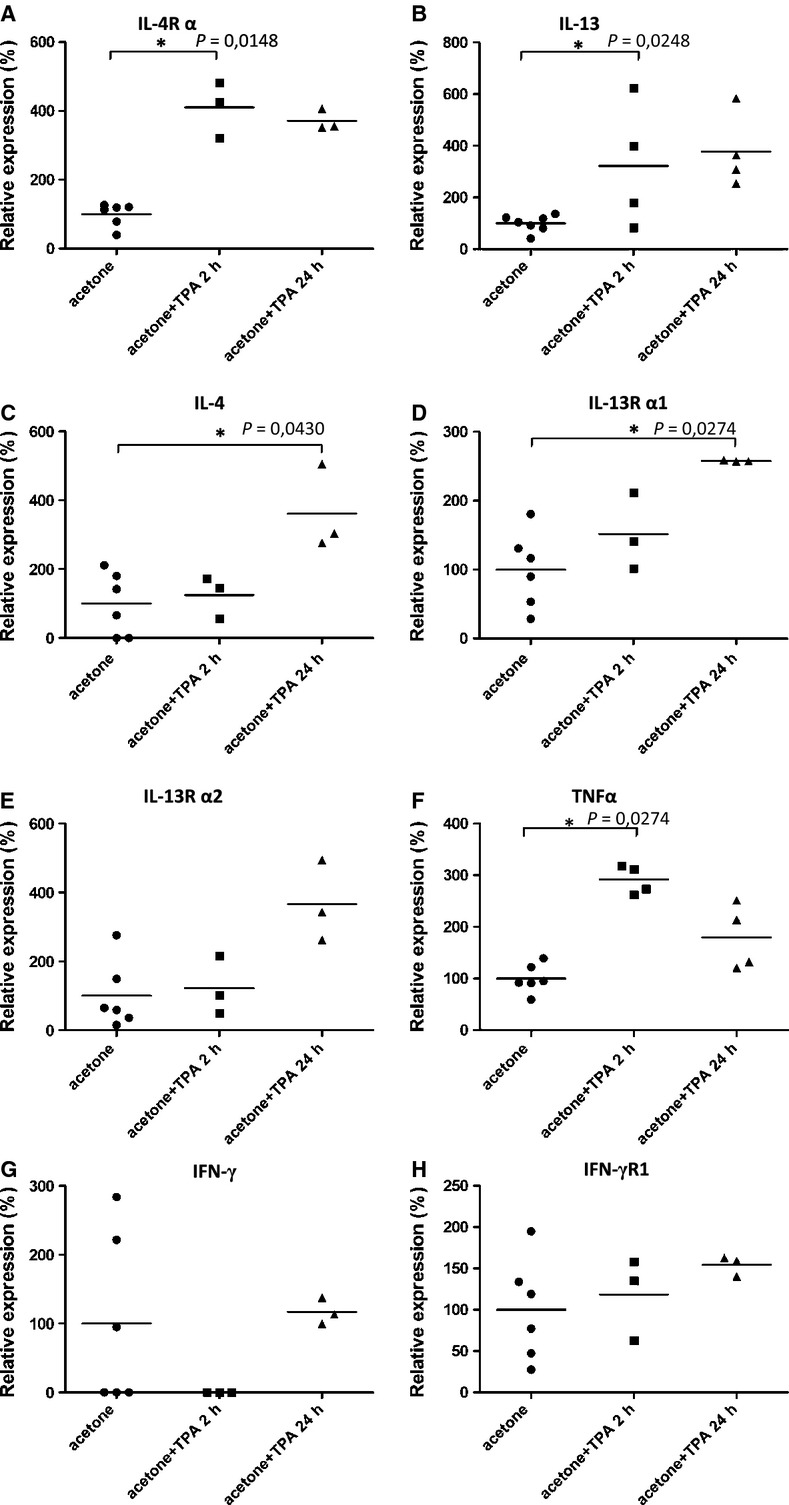
TPA treatment enhances mRNA levels of IL-4Rα, IL-13, IL-4, and IL-13Rα1. Skin of BALB/c mice (three to six females) was shaved on day 0 and treated with 4 μg TPA on day 1, 3, 5, and 7 of the experiment. The area treated was excised on day 7 either at 2 h or at 24 h after the last treatment. RNA was extracted and reverse transcribed to cDNA for quantitative real-time PCR analysis of IL-4Rα, IL-13, IL-4, IL-13Rα1, IL-13Rα2, TNF-α, IFN-γ, and IFN-γR1. Relative expression of target genes was calculated by normalization to GAPDH expression. Data presented as % relative expression of the mean in the control acetone group. Statistical significance between groups, indicated by * (*P* ≤ 0.05), was calculated using Kruskal–Wallis test. No significant difference was detected for IL-13Rα2, IFN-γ, and IFN-γR1. Filled circles: mice treated with solvent only, skin taken 24 h after last treatment; filled squares: mice treated with TPA in solvent, skin taken 2 h after last treatment; filled triangles: mice treated with TPA in solvent, skin taken 24 h after last treatment. Each symbol represents one animal. Above each panel the respective cytokine/receptor for which expression analysis was performed is indicated.

TPA application upregulates the IL-4 receptor alpha chain (IL-4Rα) expression in the skin of NMRI (inbred strain of mice) mice [20]. However, genetic background strongly affects carcinogenesis [10] and also influences whether inflammatory responses are dominated by either IL-4 or IFN-γ [21]. To study the role of IL-4 and IL-13 in skin carcinogenesis, we used the BALB/c genetic background, which tends to respond predominantly with an IL-4 driven inflammatory response. First, we tested whether IL-4Rα or other TH2-related genes (IL-4, IL-13, and IL-13 receptors) or alternatively, some TH1-related genes (IFN-γ and IFN-γR1), as well as TNF-α are upregulated in the skin of BALB/c mice upon TPA administration. To this end, we applied 4 μg TPA dissolved in acetone to a (shaved) dorsal skin area four times on consecutive days. Two hours or 24 h after the last treatment the skin was excised, RNA was extracted, transcribed into cDNA and used for quantitative PCR analysis. As shown in Figure 1A–H, IL-4Rα, IL-13, IL-4, and IL-13Rα1 were all upregulated upon TPA treatment. Importantly, upregulation of IL-4Rα and IL-13 occurred already after 2 h, whereas significant changes in the expression levels of IL-4 and IL-13Rα1 could be detected only at a later time point. TNF-α was also upregulated, which is in line with earlier published data on the role of TNF-α in this skin carcinogenesis model [22–25].

**Figure 2:**
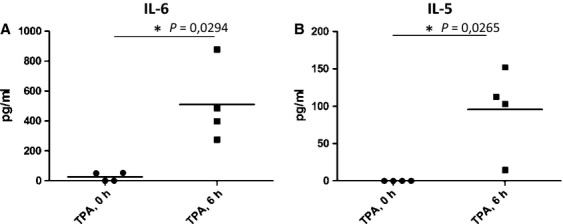
TPA treatment enhances systemic levels of IL-6 and IL-5. BALB/c mice were treated with 4 μg TPA dissolved in 100 μL acetone once. Blood samples were taken and serum prepared either directly after treatment (0 h) or 6 h after treatment. Cytokine serum levels were determined using a multiplex cytometric bead array. Shown are the total serum levels. Each symbol represents one animal. The respective treatment is indicated below the diagram. Statistical significance, indicated by * (*P* ≤ 0.05), was calculated using Mann–Whitney *U* test (two-tailed, using Gaussian approximation). Above each of the two panels the respective cytokine for which serum analysis was performed is indicated.

In parallel to investigation of the local inflammation induced by TPA (RNA expression analysis of skin tissues), we assessed the systemic cytokine response in TPA-treated mice 6 h after TPA treatment by analyzing serum levels of 10 different cytokines: IL-1α, IL-2, IL-4, IL-5, IL-6, IFN-γ, IL-10, IL-17, TNF-α, and GM-CSF (using a cytometric bead array). As shown in Figure 2A and B, we detected an increase in serum cytokine levels after TPA exposure for IL-6 (four of four mice; Fig. 2A) and IL-5 (three of four mice; Fig. 2B). For all other cytokines, there was no detectable increase in the serum. There was also no detectable increase in serum cytokine levels in the untreated group or in response to acetone treatment (data not shown).

### Enhanced papilloma formation in IL-4Rα^−/−^ mice

**Figure 3:**
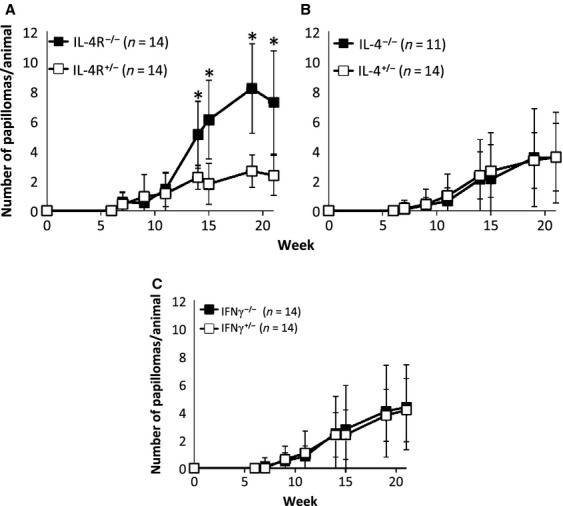
DMBA/TPA treatment enhances papilloma formation in IL-4Rα^−/−^ mice. Female mice were treated with 20 μg of DMBA. Seven days later, mice were treated with 4 μg of TPA twice a week for 20 weeks. The development of skin papillomas is shown as mean per group (±SD); numbers in parentheses indicate group size. (A) IL-4^−/−^, IL-4^+/−^; (B) IL-4Rα^−/−^, IL-4Rα^+/−^; and (C) IFN-γ^−/−^, IFN-γ^+/−^ mice. Statistical significance, indicated by * (*P* ≤ 0.05), was calculated using Mann–Whitney *U* test (two-tailed, using Gaussian approximation).

Because IL-4 and IL-4Rα were locally upregulated upon TPA treatment, we next addressed the role of IL-4 and the IL-4Rα chain in two-step carcinogenesis. Furthermore, as IL-4 and IFN-γ often counter regulate each other, we also included analysis of IFN-γ in the experiments. All of our experiments were carried out using true littermate control mice, because it has been reported that environmental factors such as transport of mice, subtle strain-specific alterations in host inflammatory responsiveness, or breeding-colony-dependent differences in commensal microflora may affect carcinogenesis [26–30]**.** Thus, we performed a standard DMBA/TPA experiment [4] by applying 20 μg DMBA and subsequently treating IL-4^+/−^, IL-4^−/−^, IL-4Rα^+/−^, IL-4Rα^−/−^, IFN-γ^+/−^, and IFN-γ^−/−^ mice for 20 weeks with TPA (4 μg three times weekly). As shown in Figure 3A, the incidence of papillomas was increased in IL-4Rα^−/−^ mice when compared to littermate control IL-4Rα^+/−^ mice and to all other experimental groups. Neither deficiency in IL-4 (Fig. 3B) nor in IFN-γ (Fig. 3C) enhanced papilloma formation when compared to heterozygous littermate control mice. A second independent experiment comparing IL-4Rα^−/−^, IL-4^−/−^, IFN-γ^−/−^, and wild-type BALB/c mice showed the same result, namely an increased papilloma incidence in IL-4Rα^−/−^ mice (data not shown).

**Figure 4:**
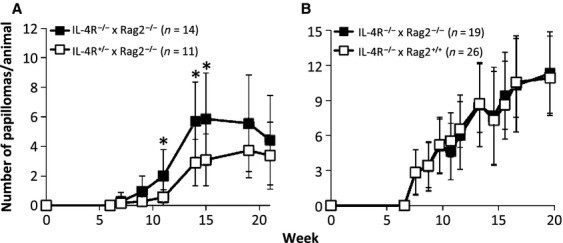
Mice lacking IL-4Rα expression also show an enhanced papilloma incidence in the absence of T cells and B cells. RAG2^−/−^/IL-4Rα^−/−^ mice and RAG2^−/−^/IL-4Rα^+/−^ mice (A) as well as RAG2^−/−^/IL-4Rα^−/−^ mice and RAG2^+/+^/IL-4Rα^−/−^ mice (B) were treated with 20 μg of DMBA. Starting day 7, mice were treated with 4 μg of TPA (twice a week) for 20 weeks. Development of skin papillomas is depicted as mean per group (±SD); numbers in parentheses indicate group size. Statistical significance, indicated by * (*P* ≤ 0.05), was calculated using Mann–Whitney *U* test (two-tailed, using Gaussian approximation). (B) These mice were not true control littermates, but were derived from the same breeding colonies kept in the same room with open cages. At week 16 and 18, three RAG2^+/+^/IL-4Rα^−/−^animals and six RAG2^−/−^/IL-4Rα^−/−^ were taken out of the experiment because of local irritation or damage to the skin after depilation.

Next, we examined if absence of T cells or B cells affected papilloma incidence with regard to the presence of IL-4Rα. To this end, we compared papilloma incidence between RAG2^−/−^/IL-4Rα^−/−^ double-knockout mice and RAG2^−/−^/IL-4Rα^+/−^ mice (Fig. 4A) as well as papilloma incidence between RAG2^−/−^/IL-4Rα^−/−^ mice and RAG2^+/+^/IL-4Rα^−/−^ mice (Fig. 4B) after DMBA/TPA treatment. This experiment showed that even in the absence of T cells or B cells, the difference in papilloma incidence between IL-4Rα-deficient and IL-4Rα-competent animals was retained although differences were less pronounced. This suggested that T cells and B cells were not the source of the protective cytokine nor was signaling in T cells or B cells necessary for the protective effect.

### While IL-13^−/−^ mice show enhanced papilloma development after DMBA/TPA carcinogenesis, tumor incidence is unaltered in IL-13^−/−^ mice as compared to IL-13^+/−^ after MCA carcinogenesis

**Figure 5:**
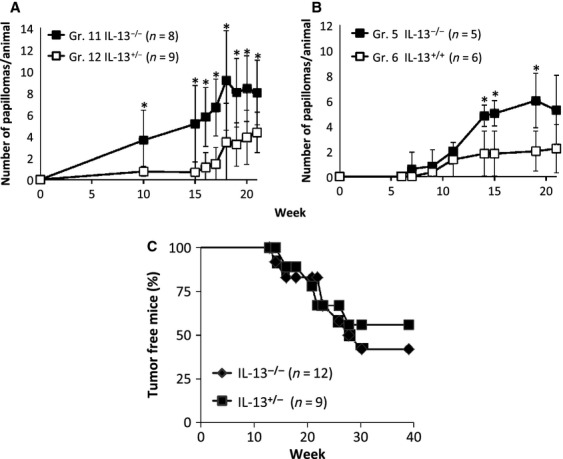
IL-13^−/−^ mice show enhanced papilloma development after DMBA/TPA carcinogenesis but unaltered tumor incidence after MCA carcinogenesis. (A) Enhanced papilloma formation in IL-13^−/−^ mice after DMBA/TPA treatment. Female mice (IL-13^−/−^; IL-13^+/−^) were treated with 20 μg of DMBA. Starting day 7, the mice were treated with 4 μg of TPA (twice a week) for 20 weeks. Development of skin papillomas is depicted as mean per group (±SD); numbers in parentheses indicate group size. Exemplary photographs documenting papilloma incidence at week 10 are shown in the Figure S1. (B) DMBA/TPA experiment as shown in (A) but using homozygous IL-13^−/−^ and IL-13^+/+^ (non littermates). Statistical significance indicated by * (*P* ≤ 0.05) was calculated using Mann–Whitney *U* test (two-tailed, using Gaussian approximation). (C) No difference in tumor incidence or latency between IL-13^−/−^ and IL-13^+/−^ mice after MCA-carcinogenesis. IL-13^−/−^ (squares) and control littermates IL-13^+/−^ (diamonds) were injected with 25 μg MCA s.c., and tumor development was observed. Numbers in parentheses indicate group size.

Because we detected an increase in papilloma incidence in IL-4Rα^−/−^ compared to IL-4^−/−^ mice (Fig. 3), we reasoned that IL-13 as the second ligand for IL-4Rα might have been responsible for this difference. Therefore, we performed a two-step skin carcinogenesis experiment in IL-13^−/−^ mice and heterozygous IL-13^+/−^ littermates (Fig. 5A) as well as homozygous IL-13^+/+^ mice (Fig. 5B) using the same protocol as mentioned above. As shown in Figure 5A and B and Figure S1, a similar, perhaps less pronounced increase in papilloma incidence was observed for IL-13^−/−^ mice as had been observed for IL-4Rα^−/−^ mice, suggesting that IL-13 signaling via IL-4Rα is responsible for the protective effect.

Previously, we had shown that IL-4 as well as B cells promote MCA carcinogenesis [26]. Because we found that IL-13 is protective in DMBA/TPA carcinogenesis, we wanted to know if IL-13 would influence MCA carcinogenesis. Therefore, we injected IL-13^−/−^ and IL-13^+/−^ mice backcrossed to the BALB/c genetic background with 25 μg of MCA. However, as shown in Figure 5C, there was no difference in tumor induction or latency (time to first tumor appearance) between IL-13^+/−^ and IL-13^−/−^ mice.

## Discussion

Various immunological factors have been previously identified that may affect two-step carcinogenesis [9, 11, 13–18, 22–25, 31]. However, it still remains unclear how exactly the inflammatory responses orchestrate tumor development in this carcinogenesis protocol. In particular, it was not known whether IL-4 or IL-13 affect DMBA/TPA carcinogenesis. In this study, we addressed this question and found that IL-4Rα and IL-13 but not IL-4 affects two-step skin carcinogenesis.

Considering that IL-4/IL-13 and IFN-γ/IL-12 counter regulate each other, our finding that IL-13 is protective is in line with the previous observation that IFN-γ and IL-12 can be tumor-promoting in DMBA/TPA carcinogenesis [15–17]. IFN-γ has been shown to be tumor-promoting in DMBA/TPA skin carcinogenesis in the majority of studies that have analyzed the role of IFN-γ [14–16, 32, 33]. However, one study reported a protective effect of IFN-γ that involved injection of plasmid DNA encoding IFN-γ into the tail vein of mice [32]. It remains unclear why IFN-γ is tumor-promoting in most DMBA/TPA carcinogenesis studies but not in this study that used mice of the BALB/c background. This may be due to the fact that BALB/c mice show an immune response that is dominated by IL-4 and do not develop a strong IFN-γ response, while other strains such as C57BL/6 mice show an IFN-γ-dominated immune response [21]. This assumption is supported by the fact that we did not observe IFN-γ RNA upregulation after TPA application to the skin of BALB/c mice.

The exact target cell that mediates the tumor protective effect upon IL-13 signaling via the IL-4Rα has yet to be defined. The fact that RAG2^−/−^/IL-4Rα^−/−^ double-knockout mice and RAG2^+/+^/IL-4Rα^−/−^ both showed increased papilloma incidence suggests that IL-13 signaling neither in T cells, nor in B cells was the main reason for enhanced papilloma incidence. Also, the source of the protective cytokine IL-13 in our experiments is not clear. However, it is unlikely that T helper cells provide IL-13 upon TPA stimulation, as studies analyzing the role of T helper cells in two-step carcinogenesis have shown that they are tumor-promoting rather than protective [14]. We consider it more likely that other innate immune cells found in the skin are the source of the protective IL-13 (such as dendritic cells [34], mast cells [35], or eosinophils [36]).

Recently, a new cell type involved in mediating TH2-cytokine-dependent skin inflammation has been identified both in humans and in mice [37]. These cells belong to group 2 innate lymphoid cells (ILC2), which produce high levels of IL-13 and IL-5 [37, 38]. This is in line with our observation that IL-13 is upregulated locally and that IL-5 is increased systemically after TPA treatment of the skin of BALB/c mice.

The contribution of cytokines to inflammation-induced cancer is complex and may not only depend on the genetic background of the mice but also on the target organ for a particular carcinogen used. One such example highlighting the importance of considering the target organ is provided in an earlier study with chemically induced colitis-associated cancer using mice of the BALB/c genetic background. In that study, the IL-4Rα signaling contributed to tumor formation rather than to being protective against tumors [39]. The outcome of carcinogenesis experiments may also be affected by environmental factors such as the composition of the microbial commensals [40]. Many environmental factors such as transport of mice, subtle strain-specific alterations in host inflammatory responsiveness, or breeding-colony-dependent differences in commensal microflora [41] may affect carcinogenesis [26, 27]. Potentially, differences in microbiota of the skin may also affect carcinogenesis, as it was reported that skin resident commensals can strongly influence the local inflammatory milieu [28]. Commensals have also been shown to affect carcinogenesis in a DMBA-driven liver tumor model [29] as well as in another liver tumorigenesis model using diethylnitrosamine and CCl_4_ [40].

One way to minimize differences in commensals would be to use control littermates [41]. We had found previously that the use of littermate control mice is essential to avoid misinterpretation of MCA carcinogenesis experiments [26, 27, 30]. However, even when using littermate controls, one cannot exclude that subtle (e.g., developmental) differences other than those directly linked to the gene defect may influence the phenotype of the mice. For example, we observed that several transgenic immunodeficient mice (including IFN-γ^−/−^ or IL-4^−/−^ mice) had slightly elevated serum levels for other cytokines than those that were genetically ablated in the respective mouse line, suggesting an altered baseline inflammatory responsiveness in these transgenic breeding colonies [27].

The systemic cytokine response after TPA application, judging by serum cytokine titers, showed upregulation of IL-5 as the only TH2 cytokine; other inflammatory cytokines that were elevated were IL-6 and TNF-α. However, neither IFN-γ nor IL-4 titers were elevated. Nevertheless, IFN-γ and IL-4 are constitutively present in untreated mice at very low levels and detectable only by the most sensitive assays [42]. Interestingly, the role of inflammation appears to differ between DMBA/TPA- and MCA-induced tumor development. For example, IL-4 enhances MCA carcinogenesis [26], but here we show that it plays no role in DMBA/TPA carcinogenesis. On the other hand, IL-13 does not affect MCA carcinogenesis but is protective in DMBA/TPA carcinogenesis. Similarly, the presence of TNF-α enhanced tumor development after the two-step carcinogenesis with either DMBA/TPA [22] or with DMBA, followed by tumor promotion with okadaic acid [25]. However, TNF-α did not affect MCA-induced tumor development [26].

The role of αβ-T cell-induced inflammation differs for both carcinogenesis protocols. While the presence of CD8^+^ T cells enhanced DMBA/TPA-induced carcinogenesis [13], T-cell-deficient mice (Nude or RAG1^−/−^) revealed almost none [30] or only a slightly enhanced susceptibility to MCA [43]. Of note is that in the absence of inflammation, T cells play no protective role by recognizing spontaneously mutated proteins, because a recent study using mutagenesis via a retrotransposition has shown that the adaptive immune system does not suppress spontaneously developing tumors [44]. The differences observed in the inflammatory response between these two models of chemical carcinogenesis (DMBA/TPA versus MCA) are supported by the observation that some mouse strains are relatively susceptible to DMBA/TPA but resistant to MCA [3, 45] (such as the 129/Sv, which respond with a high level of inflammation). In fact, IFN-γR1^−/−^ mice on the 129/Sv genetic background were more resistant to MCA than IFN-γR1^+/+^ mice on the C57BL/6 genetic background [45].

It seems that there is a marked difference between the type of inflammation that promotes MCA carcinogenesis versus that which promotes DMBA/TPA carcinogenesis. Both anti-tumorigenic (IFN-γ-mediated) and protumorigenic (IL-4-mediated) inflammatory responses appear to be involved in MCA-induced carcinogenesis. The role of IFN-γ responses for DMBA/TPA may be strain dependent, because IFN-γ is tumor-promoting in 129/Sv mice [16, 17] but plays no role in BALB/c mice. However, IL-13-driven immune responses are protective in DMBA/TPA carcinogenesis, while they do not affect MCA carcinogenesis. Involvement of IL-4Rα in DMBA/TPA carcinogenesis might have been anticipated by the observation that TPA application upregulates the IL-4Rα expression in the skin of NMRI mice [20]. We now confirmed this observation for the BALB/c genetic background and, moreover, found that IL-13Rα1 and IL-4 are both upregulated in the skin after TPA administration.

However, upregulation of IL-4 expression in the skin after TPA application seems less relevant, as IL-4-deficient mice showed the same incidence in papilloma development as IL-4-competent heterozygous littermate controls. We cannot formally exclude a gene dosage effect in heterozygous mice; wild-type (homozygous) gene levels IL-4 theoretically could influence carcinogenesis. However, in a direct comparison of heterozygous mice, the effects of IL-13 on carcinogenesis are clearly more important than those of IL-4. Our observation that IL-13 is more important than IL-4 in cutaneous inflammatory responses is in line with earlier studies [46, 47] showing that cutaneous responses are independent of IL-4 but instead depend on IL-13 in a model of atopic inflammation in the skin. One possible explanation can be due to the role of ILC2 that mediate TH2-cytokine-dependent inflammatory responses and serve as the main source of IL-13 in the skin [37, 38]. Furthermore, IL-4 and IL-13 may differentially regulate signaling in target cells by employing distinct JAK/STAT-mediated signaling pathways downstream of IL-13 and IL-4 receptor complexes, resulting in differential inflammatory responses. A recent study demonstrates that IL-4 orchestrates downstream signaling via the IL-4Rα/JAK1/STAT3/STAT6 axis, whereas IL-13 utilizes both IL-4Rα/JAK2/STAT3 and IL-13Rα1/Tyk2/STAT1/STAT6 signaling cascades to regulate expression of the target genes [48].

These results support our data and suggest that IL-13 may have unique effector functions in two-step skin carcinogenesis model, which differ from those of IL-4. Taken together, a new perspective on the differential regulation of IL-4/IL-13-mediated inflammation and the pathways previously thought to be similar should be further investigated.

## Supporting Information

**Figure S1.** Exemplary pictures of papilloma incidence in IL-13^−/−^ and in IL-13^+/−^ mice at week 10 of TPA treatment. Mice were treated with 20 μg of DMBA. Starting 7 days later, the mice were treated with 4 μg of TPA twice a week for 20 weeks. Circles indicate papillomas. Animals shown in the pictures are the same as shown in Figure 5A.
