# Supplementary figures and images for: IL-13 but not IL-4 signaling via IL-4Rα protects mice from papilloma formation during DMBA/TPA two-step skin carcinogenesis
Source: Cancer Med. 2013 Oct 22;2(6):815–25. doi: 10.1002/cam4.145 (PMC3892386; doi:10.1002/cam4.145)

## Supplementary Figure S1

IL-13<sup>-/-</sup>

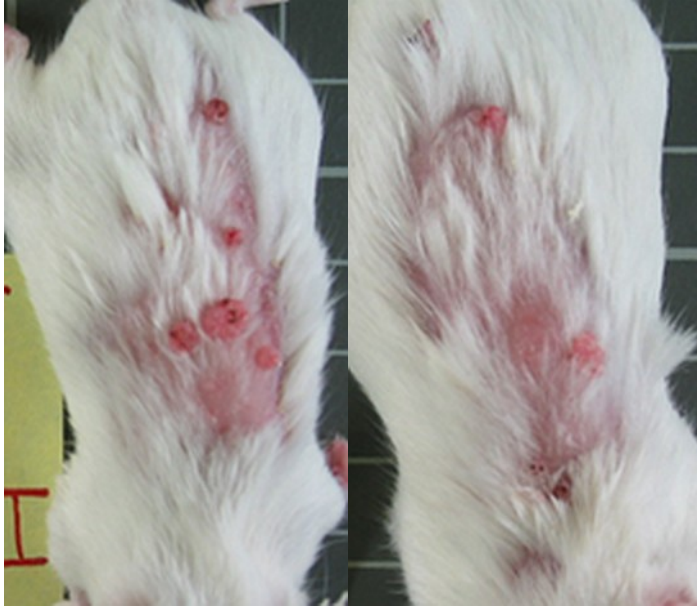

IL-13<sup>+/-</sup>

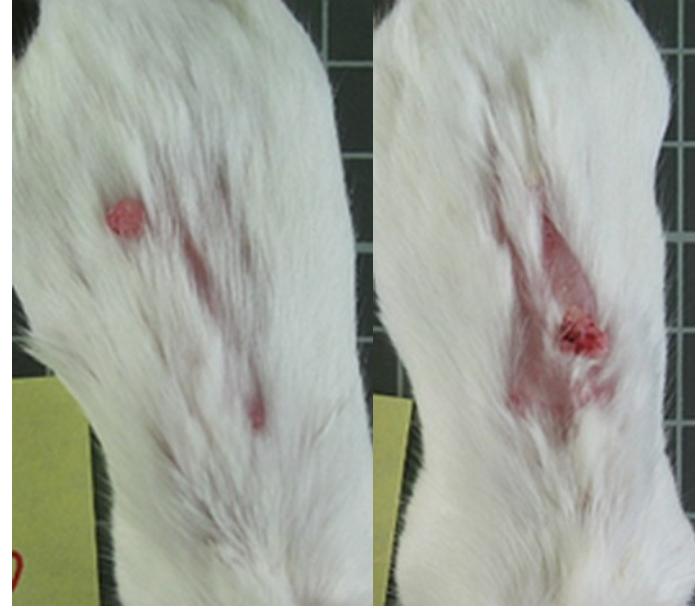

Supplement: Supplementary file 1 [file cam40002-0815-SD1.pdf]
